# Anchoring biases affect repeated scores of thermal, moisture, tactile and comfort sensations in transient conditions

**DOI:** 10.1007/s00484-018-1595-2

**Published:** 2018-08-07

**Authors:** Margherita Raccuglia, Christian Heyde, Alex Lloyd, Daniel Ruiz, Simon Hodder, George Havenith

**Affiliations:** 1 Environmental Ergonomics Research Centre, Loughborough Design School, Loughborough University, Loughborough, Leicestershire, LE11 3TU UK; 2 Adidas FUTURE Sport Science, Herzogenaurach, Germany

**Keywords:** Thermal sensation, Clothing comfort, Sensory assessments, Self-reported data

## Abstract

In this study, we addressed potential biases which can occur when sensorial scores of temperature, wetness and discomfort are repeatedly reported, in transient exercise conditions. We pointed out that, when repeatedly reported, previous sensorial scores can be set by the participants as reference values and the subsequent score may be given based on the previous point of reference, the latter phenomenon leading to a bias which we defined as ‘anchoring bias’. Indeed, the findings shown that subsequent sensorial scores are prone to anchoring biases and that the bias consisted in a systematically higher magnitude of sensation as compared to when reported a single time only. As such, the study allowed recognition, quantification and mitigation of the identified bias which can improve the methodological rigour of research studies involving assessments of sensorial data in transient conditions.

## Introduction

The direct study of human sensations and perceptions often requires the use of psychophysical scales. Psychophysical scales have been widely used to evaluate perceived exertion in physical exercise (Borg 1982), the thermal environment and thermal comfort (Backer 1948; Gagge et al. 1967; Auliciems 1981;Yang and Zhang 2008; de Dear 2011; Schweiker et al. 2017). In the context of clothing research and development, psychophysical scales have been largely used to assess thermal, moisture, haptic and comfort sensations while wearing clothing during rest and exercise conditions (Gagge et al. 1967; Fanger 1970; Hollies et al. 1979; Fanger 1986; Plante et al. 1995; Schneider et al. 1996; Fukazawa and Havenith 2009; Kaplan and Okur 2009; Jeon et al. 2011; Tang et al. 2014; Raccuglia et al. 2016, 2017b). When using psychophysical scaling, participants are asked to estimate the magnitude of a specific sensation by giving a number typically linked to a qualitative descriptor, i.e. slightly, very or extremely (Li 2001; Cardello et al. 2003). The International Standard, Ergonomics of the thermal environment—Assessment of the influence of the thermal environment using subjective judgement scales (ISO 10551:^1995^), contains a helpful guide on how to construct the scales used to measure human sensorial parameters, such as thermal sensation, thermal preference and thermal comfort. Whilst standardised guidelines (ISO 10551:^1995^) and important results (Gagge et al. 1967; Fanger 1970; McIntyre 1978; de Dear et al. 1997) have been provided for evaluations conducted in steady-state workplace conditions, they do not provide enough information regarding their repeated use over time, i.e. transient conditions, when sensations are repeatedly scored, at set intervals, over a certain period of time.

When sensorial scores are repeatedly reported in transient exercise conditions, the investigators need to decide between allowing participants to see their previous score (e.g. when scoring on sliders that remain static between scores) or preventing the use of their previous score for the following sensorial assessment (i.e. slider back to neutral point, or new fresh scale). In fact, in transient conditions, previous scores could be used by the participants as reference values. In this scenario, the subsequent score may be reported based on the previous point of reference, making the entire evaluation process relative to the past experienced sensation. The latter phenomenon can lead to a bias here defined as ‘anchoring bias’. Anchoring biases can often result from the tendency of the participants to anchor to the previously reported number (magnitude of sensation) rather than using the numbers in combination with the linked qualitative attribute of the sensation experienced. Furthermore, ‘anchoring biases’ can be the consequence of individual preconceptions. For instance, participants could make the assumption that the magnitude of a specific sensation linearly increases with the increase of exercise duration, i.e. ‘if 5 minutes ago my thermal sensation was ‘5’, now that I have run for 5 minutes longer my thermal sensation must be higher (e.g. ‘6’)’. In both scenarios where numbers (magnitude of sensation) are used as reference points, participants can lose the connection with the qualitative attribute of the experience sensation, this affecting the outcome of the research conducted. Since many researchers from multiple disciplines, i.e. neuroscience (Ackerley et al. 2012; Filingeri et al. 2014), exercise science (Christian et al. 2014; Lloyd et al. 2016), thermophysiology (Fukazawa and Havenith 2009; Schlader et al. 2011, 2013) and clothing science (Davis et al. 2017; Raccuglia et al. 2017a), use psychophysical scales to gather human sensorial and perceptual responses, it is of great importance to assess whether consecutive sensorial scores can be affected by the previous given score. In fact, recognition and quantifications of the bias can ensure scientific rigour when conducting studies involving consecutive sensorial assessments, as well as can help to find strategies to mitigate the bias. To the knowledge of the authors, it has not been demonstrated before whether, and to which extent, repeated sensorial assessments are prone to anchoring bias. As such, the aim of the current study was to assess whether there are systematic differences in sensorial scores reported at the same time point during 50 min of running exercise, in the same (separate) experimental conditions, but following a different assessment procedure, i.e. subsequent scores (every 5 min) and single score at one time point, independent of previous scores.

Specifically, since clothing strongly determines the thermal as well as comfort state of an individual (Havenith 1999, 2002; Raccuglia et al. 2016, 2017b), in this investigation, we included assessments of thermal sensation, wetness perception, stickiness sensation and wear discomfort in relation to clothing. The current findings can be of high relevance when interpreting time courses of sensorial parameters, the onset of specific sensations, or when validating thermophysiological models.

## Material and method

### Participants

Ten young (26.9 ± 3.4 years), healthy, recreationally active (strength and conditioning as well as aerobic exercises at least four times per week) male participants took part in this study. Their mean and standard deviation body mass and height were 73.5 ± 10.1 kg and 181.1 ± 8.1 cm, respectively. Participants of only Western European origin were recruited in order to reduce the variability related to differences in thermal sensitivity and preference between different ethnicities.

The experimental procedures were fully explained to the participants verbally and in writing, before obtaining written informed consent and completing a health screening questionnaire. All the experimental procedures involved were approved by the Loughborough University Ethical Committee. The study was conducted within the confines of the World Medical Association Declaration of Helsinki for medical research involving human participants.

### Garment

The experimental garment included a short sleeved, regular fitted, 100% polyester T-shirt. A fresh pre-washed (ISO 6330: 2012) T-Shirt was used for each participant and for each running trial. The T-Shirt specifications are presented in Table 1.

**Table 1:** Specifications of the experimental T-Shirt

Fibre content	Mass (g m^−2^)	Thickness (mm)	*R*_ct_ (*M*^2^ °C/W)	*R*_ef_ (m^2^ Pa/W)	Air perm (mm s^−1^)	Absorption (g m^−2^)
100% polyester	127	0.46	0.01	2.2	2088	368

*R*_*ct*_, dry thermal resistance; *R*_*ef*_, water vapour resistance; *air perm*, air permeability; *absorption*, total absorption capacity. Dry thermal resistance and water vapour resistance were measured according to BS EN ISO 11092:2014, air permeability was measured according to BS EN ISO 9137; total absorption capacity was measured according to the absorption capacity test adopted by Raccuglia et al. (2016), modified from Tang et al. (2014)

In order to ensure same regular fit between participants presenting different body dimensions, three different T-Shirt sizes were included (small, medium and large). The waist circumference of the participants was measured horizontally at waist level (where the smallest abdominal circumference occurs), while the person stands erect with the arms held slightly away from the side of the body. Three ranges of waist circumference were identified, small (68–73 cm), medium (74–79 cm) and large (80–85 cm). The circumference of each garment size, measured at the waist circumference of the participants, was taken. The latter was 90 cm for the small size, 100 cm for the medium size and 110 cm for the large size, used for small (68–73 cm), medium (74–79 cm) and large (80–85 cm) waist circumference range, respectively.

### Trials

Participants performed one pre-test and three experimental trials on different days, separated by a minimum of 24 h of rest. The pre-test involved anthropometric measurements of height, body mass (Mettler Toledo Kcc150, Mettler Toledo, Leicester, UK) and body dimensions to ensure adequate garment size used for the experimental trials. During the pre-test, participants also performed a 20-min running test on a treadmill (h/p/cosmos mercury 4.0, h/p/cosmos Sport & Medical GmbH, Nussdorf-Traunstein, Germany). During this time, the participants were asked to select the speed they could comfortably run for 1 h. The selected speed (10.1 ± 1.0 km h^−1^) was then recorded and used for the following experimental trials.

Each experimental trial involved running on the treadmill at the fixed pre-selected speed for 50 min. In the first and second experimental trials, participants were asked to report, at specific time points, one single score of thermal sensation (TS), wetness perception (WP), stickiness sensation (SS) and wear discomfort (WD). By asking to report each sensation only at one single time point, it was attempted to prevent a potential anchoring bias, therefore, the first and second experimental trials were defined as NO-ANCH1 and NO-ANCH2, respectively (Table 2). NOANCH1 and NOANCH2 only differed in the time point when the participants were asked to report the single subjective score of each investigated sensation (Table 2). In the third trial, participants were asked to score the same sensations at 5-min intervals, and due to potential anchoring biases, given by the repeated scores, this trial was defined as ANCH (Table 2).

**Table 2:** Schematic representation of the three experimental conditions

Condition	5 min	10 min	15 min	20 min	25 min	30 min	35 min	40 min	45 min	50 min
NO-ANCH1	–	–	TS	WD	SS	–	–	–	–	WP
NO-ANCH2	–	–	–	–	WP	TS	–	SS	–	WD
ANCH	TS-WD-	TS-WD-	TS-WD-	TS-WD-	TS-WD-	TS-WD-	TS-WD-	TS-WD-	TS-WD-	TS-WD-
	SS-WP	SS-WP	SS-WP	SS-WP	SS-WP	SS-WP	SS-WP	SS-WP	SS-WP	SS-WP

*NO-ANCH1*, no anchor effect trial 1; *NO-ANCH2*, no anchor effect trial 2; *ANCH*, anchor effect trial; *TS*, thermal sensation; *WD*, wear discomfort; *SS*, stickiness sensation; *WP*, wetness perception. In NO-ANCH1 and NO-ANCH2, participants were asked to report the score of TS, WD, SS and WP only once at a set time point, as reported in the table. In ANCH, participants were asked to report the score of TS, WD, SS and WP at 5-min intervals

### Experimental protocol

**Fig. 1:**
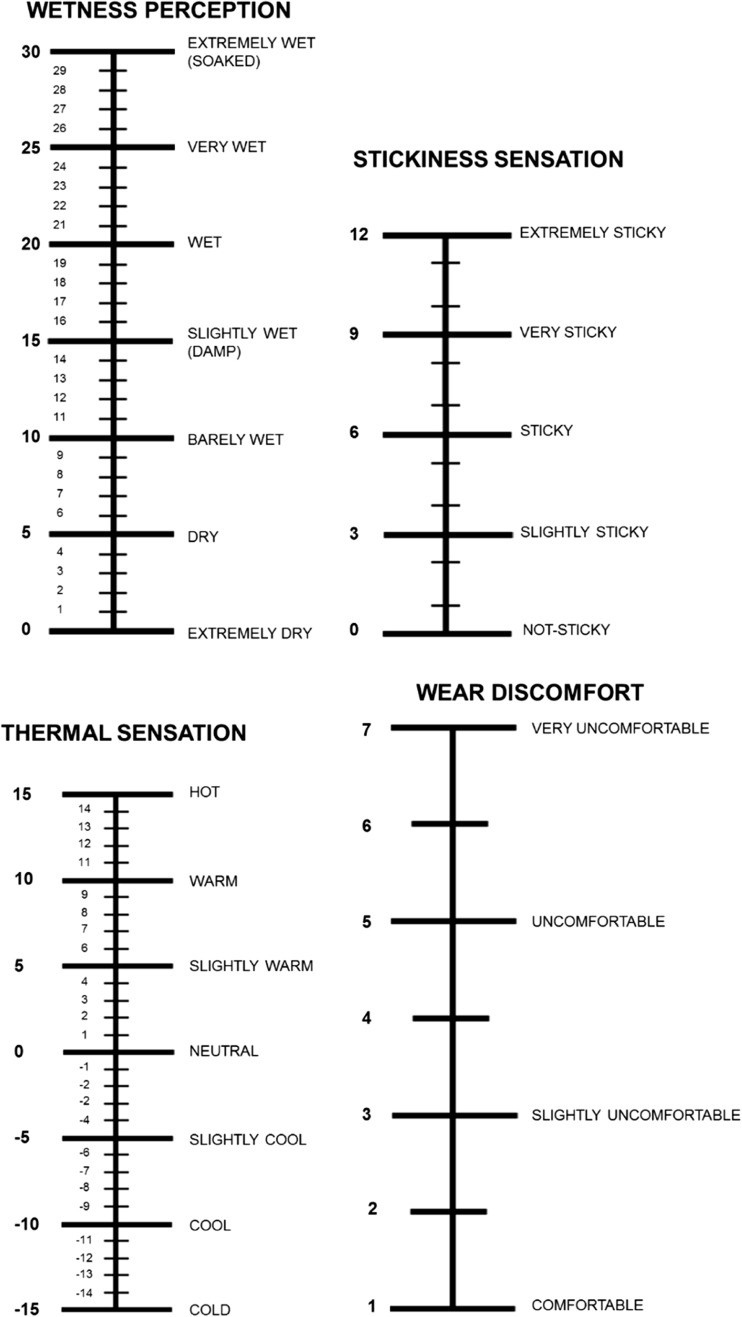
Perceptual scales. Participants scored each perceptual parameter by reporting verbally the selected number; each score was then recorded by the investigator

In each trial (NO-ANCH1, NOANCH2 and ANCH), the same garment was worn. However, to blind the participant regarding the real aim of the study, they were told that they would wear, in the three separated experimental trials, garments treated with three different moisture transfer enhancing finishes and that the purpose of the investigation was to determine the best performing one. The sequence of the two NO-ANCH trials was counterbalanced; however, to prevent any between-condition anchoring bias, the ANCH trial was always performed as last trial. During the pre-test, participants were familiarised with the psychophysical scales used in the following experimental trials (Fig. 1). During the experimental trials, each scale was displayed to the participants only when a specific score was required, in order to minimise memorisation of the previous score.

An anchoring effect could also occur between the scored sensations, e.g. if stickiness sensation is reported first, this could influence the score of the following sensations such as wetness or thermal sensation). Although this particular scenario was not the main object of the current study, it was attempted to minimise the ‘between scales’ anchoring bias by asking the participants to consider the scales independently during the scoring process.

Participants were instructed to refrain from strenuous exercise, abstain from caffeine and alcohol consumption 24 h before testing, and to keep a record of their food intake and replicate it the day before each visit. In order to maintain euhydration, they were also advised to consume 20 mL kg^−1^ body weight of water during the 2 h prior to testing. On arrival to the laboratory, participants were instrumented with iButtons (Maxim, San Jose, USA) wireless temperature loggers applied on the right side of the body at the cheek, abdomen, upper arm, lower back and back lower thigh. From these five body sites, the +mean skin temperature (*T*_sk_), sampled every minute, was estimated according to the work of Houdas and Ring (1982):

$$ \mathrm{Mean}\ {T}_{\mathrm{skin}}=\left(\mathrm{cheek}\times 0.07\right)+\left(\mathrm{abdomen}\times 0.175\right)+\left(\mathrm{upper}\ \mathrm{arm}\times 0.19\right)+\left(\mathrm{lower}\ \mathrm{back}\times 0.175\right)+\left(\mathrm{back}\ \mathrm{lower}\ \mathrm{thigh}\times 0.39\right) $$

Participants also wore a wrist-based heart rate (HR) monitor (Polar A360, Polar Electro Oy, Professorintie 5, Kempele, Finland) and HR was recorded before and during the running trials at 1-min intervals. A wrist-based monitor, rather than a chest-based strap, was used since a chest strap would have interfered with sweat transfer from the skin to the T-shirt. Following from this, semi-nude (including underwear, iButtons, and HR monitor) body mass was recorded. Subsequently, participants were provided with standard running shorts and socks, worn with their personal running shoes and were asked to use the same personal gear for the entire duration of the experiment. This period of preparation lasted approximately 20 min and allowed time for the stabilisation of HR and *T*_sk_. Participants then moved to the climatic chamber, rested standing still on the treadmill and after 10-min baseline HR was recorded. They then donned the upper garment and the running trial started. In order to prevent dehydration, the participants were allowed to drink water ad libitum during the experiment and liquid consumption was recorded. At the end of the run, participants took off the worn T-shirt and hand it over to the experimenter for measurements of post-exercise garment mass. The amount of sweat absorbed by the upper garment (SWEAT_ABS_) at the end of the running exercise was calculated as

$$ {\mathrm{SWEAT}}_{\mathrm{ABS}}\ \left(\mathrm{g}\ {\mathrm{m}}^{-2}\right)=\left[\right({w}_{\mathrm{wet}}-{w}_{\mathrm{dry}}\Big]/ SA $$

where:

*w*_wet_: Garment wet weight (g)
*w*_dry_: Garment dry weight (g)
*SA*: Garment surface area (m^2^)

Participants took off shorts, socks and shoes; towelled their skin (this took ~ 2 min) and post-exercise semi-nude body mass was recorded. Sweat production was calculated based on the weight change of each participant (gross sweat loss, GSL), corrected for liquid intake, and reported in grams per body surface area (g m^−2^), according to:

$$ \mathrm{GSL}\ \left(\mathrm{g}\ {\mathrm{m}}^{-2}\right)=\left[{w}_{b1}-\left({w}_{b2}-\mathrm{liquid}\right)\right]/ SA $$

where:

*w*_*b*1_: Body mass at the start of the experiment (g)

The experiment was conducted in a climatic chamber maintained at 27.3 ± 0.2 °C, 49.9 ± 5.6% relative humidity and wind speed corresponding to 75% of the individual running speed (9.5 ± 6.2 m s^−1^).

### Perceptual measurements

Wetness perception, stickiness sensation (SS), thermal sensation (TS) and wear discomfort (WD) were scored by the participants using psychophysical scales (Fig. 1). Wetness perception was scored using a unipolar scale ranging from 0 (extremely dry) to 30 (extremely wet) (Raccuglia et al. 2016, 2017b). Stickiness sensation was scored using a 12-point unipolar scale (0 not sticky, 12 extremely sticky) (Raccuglia et al. 2017b). Thermal sensation was scored using a bipolar scale ranging from − 15 cold to 15 hot (Raccuglia et al. 2016). Finally, the increase in wear discomfort was scored using a unipolar scale, ranging from 1 comfortable to 7 very uncomfortable (Fig. 1).

## Statistics

The dependent variables were as follows: HR, *T*_sk_, GSL (physiological) SWEAT_*ABS*_, wetness perception, stickiness sensation, thermal sensation and wear discomfort (sensorial). Data were tested for normality of distribution and homogeneity of variance with Shapiro-Wilk and Levene’s tests, respectively. One-way repeated measures ANOVA tests were performed to assess differences in HR, *T*_sk_, GSL and SWEAT_*ABS*_ between the trials (NO-ANCH1, NO-ANCH2 and ANCH). Non-parametric Wilcoxon signed-rank test were conducted to assess differences in wetness perception, stickiness sensation and wear comfort between the trials (NO-ANCH1, NO-ANCH2 and ANCH) at the selected time points (Table 2). In all analyses, *p* < 0.05 was used to establish significant differences. Data are reported as mean ± standard deviation. Statistical analysis was performed using the software IBM SPSS Statistics version 23 (IBM, Chicago, USA).

## Results

### Physiological measurements

**Fig. 2:**
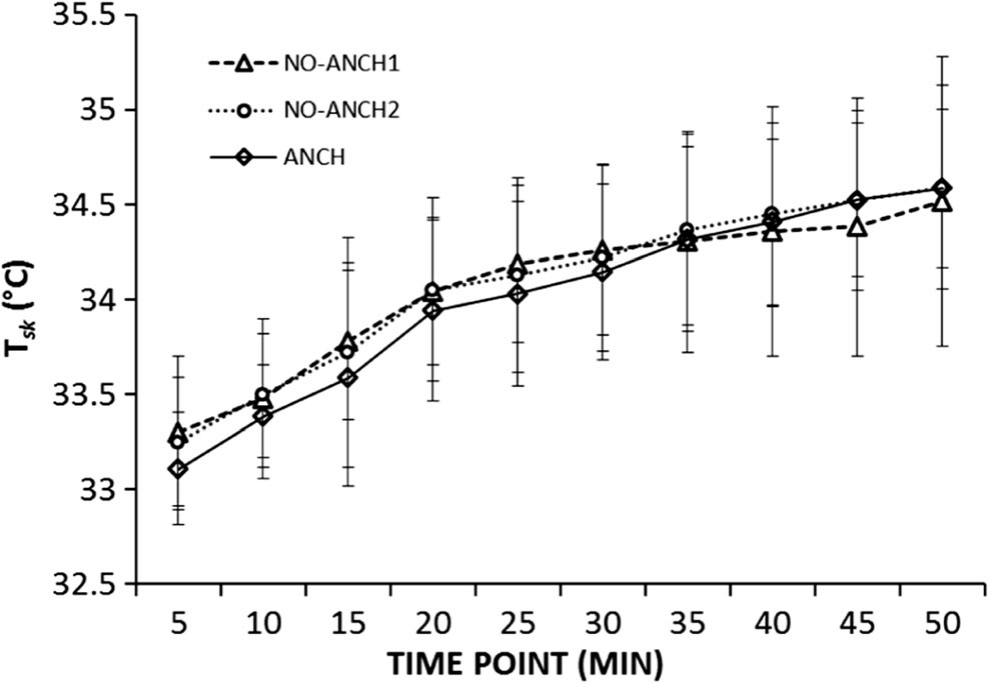
Time course of mean skin temperature (*T*_sk_) recorded at 1-min interval and sampled every 5 min in the NO-ANCH 1 and NO-ANCH2 trials (no anchoring effect) as well as in the ANCH (anchoring effect) trial

There were no significant differences in the amount of total sweat produced (GSL) (*p* = 0.54), heart rate (HR) (*p* = 0.48) and amount of sweat absorbed by the garment (SWEAT_*ABS*_) (*p* = 0.76) between the three experimental trials (NO-ANCH1, NO-ANCH2, ANCH) (Table 3). Mean *T*_sk_, measured at 1-min interval and sampled over 5-min intervals during the whole duration of the run (Fig. 2), was not significantly different between the three trials (NO-ANCH1, NO-ANCH2 and ANCH) at any time point (*p* > 0.05). Therefore, the three trials provided the same thermal load and thermoregulatory responses for each participant which resulted in same level of garment physical wetness. Based on this, it can be inferred that potential systematic differences in sensorial scores can only be due to the methodology in which the participants were asked to report the sensorial scores, i.e. at 5-min intervals (ANCH) or at one single time point (ANCH1 and ANCH2).

**Table 3:** Physiological responses across the three experimental trials

Condition	GSL (g m^−2^)	SWEAT_ABS_ (g m^−2^)	HR (bmp)
NO-ANCH1	519.6 ± 89.1	94.0 ± 56.5	152 ± 11
NO-ANCH2	514.8 ± 94.1	98.3 ± 66.1	156 ± 9
ANCH	514.7 ± 96.0	96.8 ± 62.2	154 ± 10

*NO-ANCH21*, no anchoring effect trial 1; *NO-ANCH2*, no anchor effect trial 2; *ANCH*, anchoring effect trial; *GSL*, gross sweat loss; *SWEAT*_*ABS*_, amount of sweat absorbed by the upper garment at the end of the running exercise; *HR*, heart rate at the end of the run (at 50 min). Data are presented as mean ± standard deviation

### Sensorial scores

**Fig. 3:**
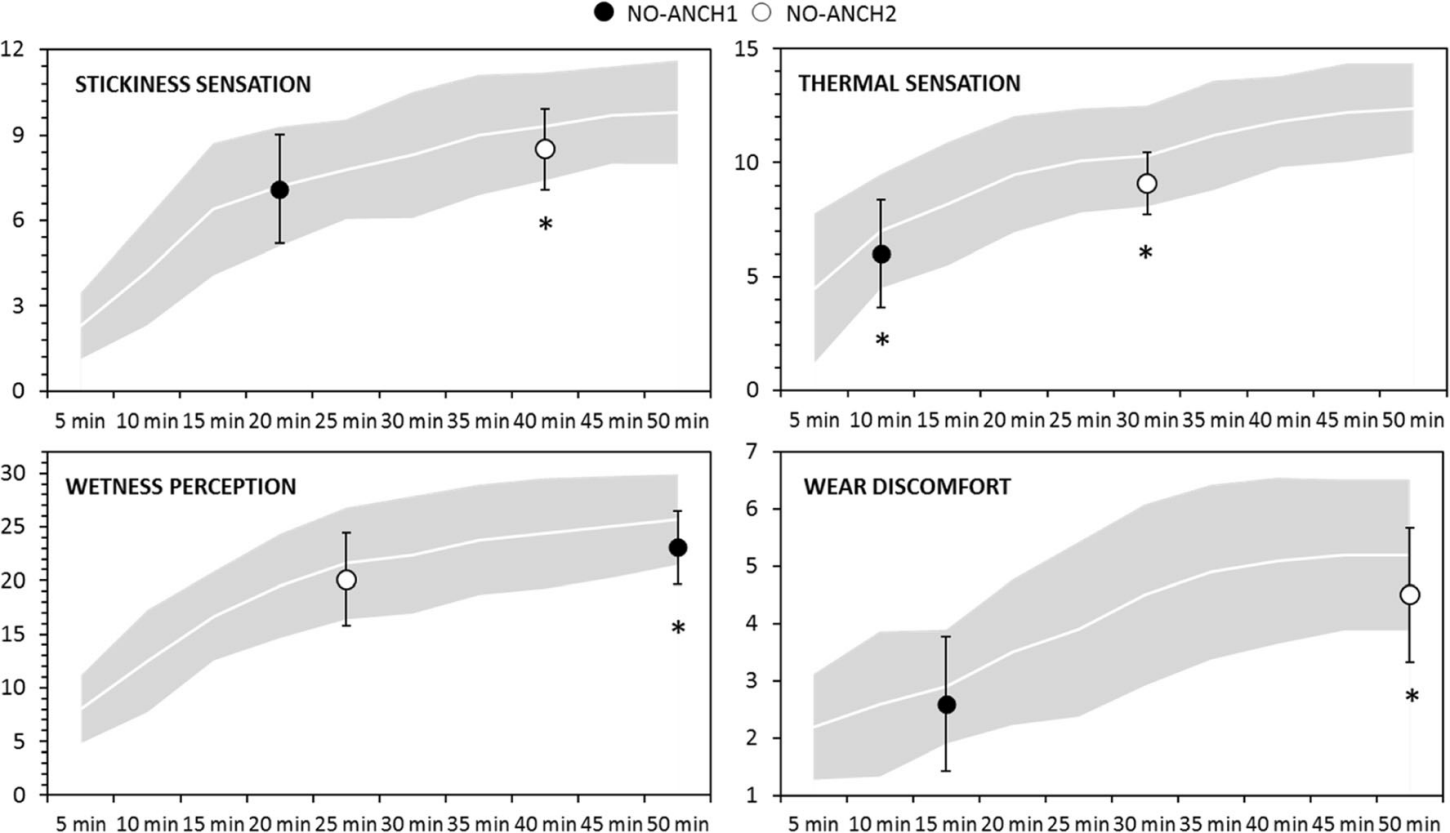
Reported perceptual scores of stickiness sensation, thermal sensation, wetness perception and wear discomfort. The solid line represents the perceptual scores reported at 5-min intervals in the ANCH trial, and the grey area indicates the corresponding standard deviation. The black and white circles represent means of the single time-point sensorial scores reported in NO-ANCH1 and NO-ANCH2 trial, respectively. *Significant differences (*p* < 0.05) between NO-ANCH1 and ANCH as well as between NO-ANCH2 and ANCH at specific time points

For clarity, the duration of the running trials is divided in two parts. The first part of the run ranges from 5 to 25 min, the second one from 25 to 50 min. The results show that the scores reported at 5-min intervals in ANCH were always higher than the single time-point scores obtained from NO-ANCH1 and NO-ANCH2 (Fig. 3, Table 4).

**Table 4:** Sensorial scores in ANCH, NO-ANCH1 and NOANCH2, at same time points (Table 2)

Sensorial score	ANCH	NO-ANCH1	ANCH	NO-ANCH2
Wetness perception	25.7 ± 4.2*	23.1 ± 3.4	22.2 ± 4.2	20.1 ± 4.3
Thermal sensation	7 ± 2.5*	6 ± 2.3	10.3 ± 2.2*	9.1 ± 1.4
Stickiness sensation	7.2 ± 2.1	7.1 ± 1.9	9.3 ± 1.9*	8.5 ± 1.4
Wear discomfort	2.9 ± 1.0	2.6 ± 1.2	5.2 ± 1.3*	4.5 ± 1.8

*NO-ANCH 1*, no anchoring effect trial 1; *NO-ANCH2*, no anchoring effect trial 2; *ANCH*, anchoring effect trial. *Significant differences (*p* < 0.05) between ANCH and NO-ANCH1 and between ANCH and NO-ANCH2

In the first part of the running trial (5 to 25 min), only sensorial scores of thermal sensation were significantly higher (*p* = 0.017, *z* = − 2.43) in ANCH, compared to NO-ANCH1 (Fig. 2, Table 4), whereas scores of stickiness sensation, wetness perception and wear discomfort did not reach significance (*p* = 0.77, *z* = − 0.33 stickiness; *p* = 0.06, *z* = − 1.89 wetness; *p* = 0.2, *z* = − 1.34 discomfort). In the second part of the running trial (25 to 50 min), all the investigated sensations (wetness perception, thermal sensation, stickiness sensation and wear discomfort) were significantly higher (*p* < 0.05) in the ANCH trial compared to the NO-ANCH1 and NO-ANCH2 (*p* = 0.02, *z* = − 2.36 thermal; *p* = 0.03, *z* = − 2.13 stickiness; *p* = 0.01, *z* = 2.49 wetness; *p* = 0.02, *z* = 2.33 discomfort) (Fig. 3, Table 4).

The magnitude of the bias did not differ significantly between the first and the second part of the running trial for thermal sensation (*p* = 0.64), stickiness sensation (*p* = 0.55) wetness perception (*p* = 0.45) and wear discomfort (*p* = 0.168).

## Discussion

The focus of this study was to assess whether and to which extent ‘anchoring biases’ occur when the magnitude of specific sensations is repeatedly reported in exercise. Our main finding was that scores of thermal, stickiness, wetness and discomfort sensation are significantly higher when subsequently reported at set intervals (every 5 min), as compared to single time-point scores. These findings show that subsequent sensorial scores are prone to anchoring biases and that the bias consists in a systematically higher estimated magnitude of a particular sensation as compared to when reported a single time only.

### Psychophysical scaling and past anchoring bias

Psychophysical scaling gathers direct information regarding the subjective experience that a person has in a specific environment. Sensorial data obtained from psychophysical scales can be considered as self-reported data. The common assumptions made with self-reported sensorial data is that the data represent an accurate, unbiased reflection of what is being measured (Dodd-McCue and Tartaglia 2010). However, the validity of subjective data (obtained using psychophysical scales) is often questioned. Particularly, self-reported data can introduce biases, mainly originated from the subjective nature of the human participants. Biases can affect the quality of the measurement, causing inaccuracy or lack of precision of the research. Participants can be inaccurate or cause biased responses for numerous reasons. For instance, participants may want to be consistent in their responses, rather than focusing on the question asked. In other cases, participants might be concerned on how their responses can affect the opinion that others (investigator) can have of them (Mick 1996). In the literature, several biases related to self-reported data are examined (Krosnick 1999; Polit and Beck 2004; Dodd-McCue and Tartaglia 2010). However, there is not information available regarding potential biases occurring when sensorial data are repeatedly reported over time in exercise; the latter being the focus of the current study. Specifically, here, we considered over-time assessments of temperature, stickiness, wetness and discomfort, in relation to clothing during exercise. We hypothesised that, when sensorial data are repeatedly reported over time, the previous provided score might serve as reference for determining the subsequent response. This phenomenon might sound similar to the so-called halo effect (Polit and Beck 2004), in which the individual assessment of a certain object triggers the pattern of the following response. However, in the current study, we hypothesised that the previous reported data is set by the participants as reference to intentionally report a subsequent response different from the one previously provided. This could be simply based on the assumption that over time the magnitude of a certain sensation must change, as exercise time/intensity increases. To test this hypothesis, in the current investigation, sensations of temperature, wetness, stickiness and wear discomfort were measured at set single time points, over 50 min of running exercise, and compared to the response provided (in a separated trial) at the same time point, but as part of subsequent measurements, i.e. every 5 min. In line with our hypothesis, repeated measurements significantly differed from single time-point measurements. Specifically, systematically higher scores were identified when provided multiple times as compared to the single-time scores (Fig. 3). Despite the lack of significant differences, anchoring biases tended to be higher in the second part of the run as compared to the first part (Fig. 3), suggesting that the magnitude of anchoring biases can increase over time. Differences in sensorial responses between ANCH and NO-ANCH condition were mainly affected by the scoring procedure adopted (i.e. repeated versus single time scores). In fact, physiological parameters of mean skin temperature, heart rate and gross sweat loss were not significantly different between the experimental trials. Core temperature, usually measured rectally or via an oesophageal probes, was not measured to avoid potential discomfort which could interfere with the sensorial responses of temperature, wetness, stickiness and wear discomfort. Additionally, as ANCH was always performed as last trial, it was important to exclude any order effect on the sensations investigated. NO-ANCH1 and NO-ANCH2 were performed in balanced order, therefore five participants performed NO-ANCH1 as the first trial and five other participants performed NO-ANCH1 as the second trial. There was not tendency to report higher or lower sensations in NO-ANCH1 performed as the second trial as compared to NO-ANCH1 performed as the first trial (the same was observed in NO-ANCH2), this suggesting that there was no order effect in the investigated sensations. As such, even though ANCH was always performed as last trial (in to prevent any between-condition anchoring bias), it can be excluded that this impacted the magnitude of the reported sensations in ANCH.

According to these findings, here, we propose that when reported in a repeated evaluation process (at set intervals, over time, in exercise), the previous score is set as reference to intentionally provide a greater subsequent score. This can be affected by the tendency of the participants to anchor to previous numbers rather than using the qualitative attribute as reference. In fact, when using numbers rather than the attributes, participants can lose the link with the actual magnitude of the sensation experience. Furthermore, anchoring biases can occur as result of a biased individual’s preconception that the progression of exercise time/intensity is accompanied by concomitant increases of the magnitude of a specific sensation, even if a greater sensation is not necessarily perceived. In cognitive psychology, this phenomenon can be recognised as schema. Schemas are stored in long-term memory and include knowledge about events and consequence of events (scripts) (Eysenck and Keane 2010). Schemas allow us to form expectations, as in this case, as exercise progresses, participants expect to have higher body temperature and sweating responses, which can influence the magnitude of the related sensation reported.

Anchoring biases represent limiting factors in studies aiming to identify critical threshold values, i.e. onset of fatigue or discomfort, and associated sensations of temperature and wetness. However, this kind of bias might not represent an issue if the aim of the research is to simply discriminate between two or more items (i.e. garments). In fact, in this type of research, it is important to assess the discriminatory power between items but the magnitude of the score per se is not relevant. However, anchoring biases can lead to a ‘ceiling effect’. In exercise, this can occur when the score becomes progressively higher over time, to the point where the maximum value on the scales is prematurely reached before the end of the evaluation process, i.e. when assessing perceived exertion (Borg 1982) induced by different exercise protocols/stimulations. As such, once the highest value of a particular scale is achieved, discriminations between items cannot be made. On the contrary, based on the current findings, in other settings, having a previous score value as reference can help to make more accurate judgments, meaning that the tendency to anchor to the previous score not always lead to a bias. This can occur when participants have to make estimations regarding the magnitude of absolute, continuous values, i.e. estimations of time progression, for instance.

These results are also relevant for the validation and improvement of thermophysiological models. In fact, given that anchoring biases causes higher magnitude of thermal sensation, in cases where thermal sensation scores are used together with other physiological parameters to estimate strain levels, for instance, this can lead to the development of inaccurate important guidelines, for instance work-rest cycles or underestimations of critical threshold values, i.e. time to heat exhaustion. In the context of physical work activities performed in the heat, underestimations of such outcomes could negatively impact productivity and general economy.

Finally, anchoring biases could contribute to inaccuracies of thermal comfort models. For instance in the model developed by Fiala et al. (2012), thermal comfort predictions were developed by correlating series of experimentally observed overall thermal sensation scores with dynamically predicted variables of the bodily thermal states. These served as a basis for developing the dynamic thermal sensation, DTS using the seven-point ASHRAE scale running from − 3 for cold to + 3 for hot (Fiala et al. 2003). Nevertheless, if the current prediction is used to estimate thermal sensation and related (dis)comfort in more extreme condition, i.e. hot/warm exercise condition, anchoring bias could lead to variations of the predicted thermal/comfort sensation.

The current study has shown successfully that anchoring biases occur when certain sensations/perceptions are reported over time and in transient exercise conditions. Results from our laboratory have recently indicated that assessment of stickiness sensations and wetness perception that are independent are the two sensations that are considered separately (Raccuglia et al. 2018). Nevertheless, assessing whether other sensorial scores are also independent or whether they influence each other’s, e.g. higher stickiness sensation might trigger higher wetness sensation, is a topic that requires further investigations.

### Recognition and mitigation

Self-reported data are one of the most effective and appropriate way to gather information regarding the magnitude of specific sensations (Dodd-McCue and Tartaglia 2010), in our case, temperature, wetness, tactile and discomfort, while wearing clothing during exercise. When conducting research involving sensorial subjective data, it is common to pay attention to sample size, research design and statistical analysis, to improve methodological rigour. An additional factor needs to be considered by the researcher and this is the recognition of the potential bias, which is intrinsic of sensorial self-reported data. In fact, while it seems unrealistic to completely prevent and avoid biases, it is possible to address some of them before and during the data collection, to obtain a more precise interpretation of the study results. Particularly, in this study after recognition of the anchoring bias, the magnitude of the bias was quantified (Fig. 3, Table 4).

Mitigation of the bias is the second step which should be considered by the researcher. It is crucial to identify the factors that can make the subjective sensorial response prone to bias. In the current study, to mitigate the past anchoring bias, specific strategies were adopted. For instance, while in some research set-ups, it is common to show to the participants the scales during the entire duration of the experimental trial; in this study, each scale was displayed only when a specific sensorial score was required. As such, memorisation and habituation to the scales, which could contribute to anchoring biases, were prevented. Anecdotally, in the trial requiring repeated sensorial scores, some participants asked the investigator to remind them their previously reported score before giving the next one. This event clearly demonstrates that participants tend to ‘anchor’ to the previous score and to set it as reference for the following one. Therefore, an additional strategy adopted to mitigate the bias was to negate reminders of the previous score and encourage the participant to focus and keep the connection with the sensation experience at that specific time point, using numbers as well as qualitative sensory attributes. Finally, another strategy which could further mitigate anchoring biases consists of reducing the frequency in which sensorial scores are asked to be reported. A longer interval between two consecutive scores could attenuated memorisation, and in this way, participants become less prone to relate the following score to the previous given one.

## Conclusion

The aim of the current study was to investigate the use of psychophysical scales in transient conditions, when the magnitude of a sensation is repeatedly scored, at set intervals, over a certain period of exercise time. Repeated sensorial scores were compared with single time-point score, assessed in the same exercise and environmental conditions. This investigation demonstrated that repeated scores of thermal sensation, wetness perception, stickiness sensation and wear discomfort, in relation to clothing, during exercise, are significantly higher than single time-point scores. This confirms our hypothesis that when repeatedly reported in transient exercise conditions, sensorial scores are prone to anchoring biases. In particular, due to the tendency to anchor to the previous number, rather than the sensorial descriptor, and to a bias preconception that sensations are linearly related to exercise progression, participants tend to intentionally give a score which is systematically higher than a score given in the same situation but a single time only. Although in the current study, the sensations investigated where related to clothing, we speculate that same past anchoring biases can occur when assessing other subjects, e.g. thermal environment, vision, noises, fatigue or exercise. While complete abolishment of anchoring biases seems unrealistic, we recognised and quantified the bias which is important for the interpretation of future study’s results. Finally, we provided strategies to mitigate the bias, in order to improve the rigour of research involving sensorial self-reported data.

## References

[CR1] Ackerley R, Olausson H, Wessberg J, McGlone F (2012). Wetness perception across body sites. Neurosci Lett.

[CR2] Auliciems A (1981). Towards a psycho-physiological model of thermal perception. Int J Biometeorol.

[CR3] Backer S (1948). The relationship between the structural geometry of a textile fabric and its physical properties: I: literature review. Text Res J.

[CR4] Borg GA (1982). Psychophysical bases of perceived exertion. Med Sci Sports Exerc.

[CR5] Cardello AV, Winterhalter C, Schutz HG (2003). Predicting the handle and comfort of military clothing fabrics from sensory and instrumental data: development and application of new psychophysical methods. Text Res J.

[CR6] Christian RJ, Bishop DJ, Billaut F, Girard O (2014). The role of sense of effort on self-selected cycling power output. Front Physiol.

[CR7] Davis JK, Matt LC, Allen KE (2017). Influence of clothing on thermoregulation and comfort during exercise in the heat. J Strength Cond Res.

[CR8] de Dear R (2011). Revisiting an old hypothesis of human thermal perception: alliesthesia. Build Res Inf.

[CR9] de Dear R, Brager GS, Cooper D (1997) Developing an adaptive model of thermal comfort and preference. Final Report-Final Report (ASHRAE RP-884)

[CR10] Dodd-McCue D, Tartaglia A (2010). Self-report response bias: learning how to live with its diagnosis in chaplaincy research. Chaplaincy Today.

[CR11] Eysenck MW, Keane MT (2010). Cognitive psychology: a student’s handbook.

[CR12] Fanger PO (1970). Thermal comfort. Analysis and applications in environmental engineering.

[CR13] Fanger PO (1986). Thermal environment — human requirements. Environmentalist.

[CR14] Fiala D, Lomas KJ, Stohrer M (2003). First principles modelling of thermal sensation responses in steady state and transient boundary conditions. ASHRAE Trans.

[CR15] Fiala D, Havenith G, Bröde P, Kampmann B, Jendritzky G (2012). UTCI-Fiala multi-node model of human heat transfer and temperature regulation. Int J Biometeorol.

[CR16] Filingeri D, Fournet D, Hodder S, Havenith G (2014). Why wet feels wet? A neurophysiological model of human cutaneous wetness sensitivity. J Neurophysiol.

[CR17] Fukazawa T, Havenith G (2009). Differences in comfort perception in relation to local and whole body skin wettedness. Eur J Appl Physiol.

[CR18] Gagge AP, Stolwijk JA, Hardy JD (1967). Comfort and thermal sensations and associated physiological responses at various ambient temperatures. Environ Res.

[CR19] Havenith G (1999). Heat balance when wearing protective clothing. Ann Occup Hyg.

[CR20] Havenith G (2002). Interaction of clothing and thermoregulation. Exog Dermatol.

[CR21] Hollies NRS, Custer AG, Morin CJ, Howard ME (1979). A human perception analysis approach to clothing comfort. Text Res J.

[CR22] Houdas Y, Ring E (1982). Human body temperature - its measurement and regulation.

[CR23] International Organization for Standardisation ISO 10551:1995 (1995) Ergonomics of the thermal environment -- Assessment of the influence of the thermal environment using subjective judgement scales. ISO, Geneva

[CR24] International Organization for Standardization ISO 6330:2012 Textiles -- Domestic washing and drying procedures for textile testing. ISO, Geneva

[CR25] Jeon E, Yoo S, Kim E (2011). Psychophysical determination of moisture perception in high-performance shirt fabrics in relationtosweating level. Ergonomics.

[CR26] Kaplan S, Okur A (2009). Determination of coolness and dampness sensations created by fabrics by forearm test and fabric measurements. J Sens Stud.

[CR27] Krosnick JA (1999). Survey research. Annu Rev Psychol.

[CR28] Li Y (2001). The science of clothing comfort. Text Prog.

[CR29] Lloyd A, Raccuglia M, Hodder S, Havenith G (2016). Interaction between environmental temperature and hypoxia on central and peripheral fatigue during high-intensity dynamic knee extension. J Appl Physiol.

[CR30] McIntyre DA (1978) Seven point scales of warmth. Build Serv Eng Res Technol 45:215–226

[CR31] Mick DG (1996). Are studies of dark side variables confounded by socially desirable responding? The case of materialism. J Consum Res.

[CR32] Plante AM, Holcombe BV, Stephens LG (1995). Fiber hygroscopicity and perceptions of dampness: part I: subjective trials. Text Res J.

[CR33] Polit DF, Beck CT (2004). Nursing research: principles and methods, seventh.

[CR34] Raccuglia M, Hodder S, Havenith G (2016). Human wetness perception in relation to textile water absorption parameters under static skin contact. Text Res J.

[CR35] Raccuglia M, Hodder S, Havenith G (2017). Human wetness perception: from skin to clothing. Med Sci Sports Exerc.

[CR36] Raccuglia Margherita, Pistak Kolby, Heyde Christian, Qu Jianguo, Mao Ningtao, Hodder Simon, Havenith George (2017). Human wetness perception of fabrics under dynamic skin contact. Textile Research Journal.

[CR37] Raccuglia M, Sales B, Heyde C, Havenith G, Hodder S (2018). Clothing comfort during physical exercise – determining the critical factors. Appl Ergon.

[CR38] Schlader ZJ, Simmons SE, Stannard SR, Mündel T (2011). The independent roles of temperature and thermal perception in the control of human thermoregulatory behavior. Physiol Behav.

[CR39] Schlader ZJ, Perry BG, Jusoh MRC, Hodges LD, Stannard SR, Mündel T (2013). Human temperature regulation when given the opportunity to behave. Eur J Appl Physiol.

[CR40] Schneider AM, Holcombe BV, Stephens LG (1996). Enhancement of coolness to the touch by hygroscopic fibers: part I: subjective trials. Text Res J.

[CR41] Schweiker M, Fuchs X, Becker S, Shukuya M, Dovjak M, Hawighorst M, Kolarik J (2017). Challenging the assumptions for thermal sensation scales. Build Res Inf.

[CR42] Tang KPM, Kan CW, Fan JT (2014). Assessing and predicting the subjective wetness sensation of textiles: subjective and objective evaluation. Text Res J.

[CR43] Yang W, Zhang G (2008). Thermal comfort in naturally ventilated and air-conditioned buildings in humid subtropical climate zone in China. Int J Biometeorol.

